# Rural-Urban Disparities in the Cognitive Returns to Education Among Chinese Older Adults: A Longitudinal, Causal Robustness, and Mechanism Analysis

**DOI:** 10.21203/rs.3.rs-10458393/v1

**Published:** 2026-07-29

**Authors:** songwei ru, xinxin yan, na guo, huimin liu

**Affiliations:** Aerospace Center Hospital; Aerospace Center Hospital; Aerospace Center Hospital; Aerospace Center Hospital

**Keywords:** Cognitive function, Education, Rural-urban disparity, Older adults, CHARLS, Resource substitution

## Abstract

**Background:**

Educational attainment is a key protective factor against late-life cognitive decline, yet whether its cognitive returns are heightened in resource-deprived rural environments remains controversial. We investigated whether rural-urban residency moderates the association between education and cognitive function among older Chinese adults.

**Methods:**

Using data from the China Health and Retirement Longitudinal Study (CHARLS; N = 15,646), cognitive function was operationalized via episodic memory (word recall, 0–20). Hierarchical ordinary least squares (OLS) and linear mixed-effects models estimated cross-sectional and longitudinal Education × Rural interactions. Propensity score matching (PSM), E-value sensitivity analysis, and an expanded nine-mediator framework evaluated causal robustness, dimension specificity, and underlying mechanisms.

**Results:**

The Education × Rural interaction was positive and statistically significant (β = 0.108, p < 0.001), demonstrating larger cognitive returns of education for rural residents. This advantage remained consistent across gender and age subgroups and expanded longitudinally over time (β = 0.054, p < 0.001). PSM confirmed directional stability (β = 0.055, p = 0.037) despite a 48% attenuation. E-value analysis indicated robust main effects (E = 2.21) alongside a more sensitive interaction contrast (E = 1.20). Dimension-specificity tests revealed independent spatial and economic resource substitution, with ~ 95% of the interaction operating via direct cognitive reserve building rather than measurable behavioral or social pathways.

**Conclusion:**

Educational attainment yields stronger cognitive protective returns in rural contexts, lending empirical support to the resource substitution hypothesis. However, the sensitivity of the interaction contrast to unmeasured confounding warrants careful interpretation when setting policy expectations.

## 1. Introduction

Global population aging has led to a rapid increase in the prevalence of cognitive impairment and dementia, imposing substantial health and socioeconomic burdens worldwide [1, 2, 15]. The global count of individuals living with dementia is projected to reach 139 million by 2050, with the steepest increases concentrated in low- and middle-income countries [16, 17]. In China alone, over 15 million adults aged 60 and older are living with dementia, accounting for roughly one-quarter of the global burden [18]. Although cognitive aging is a complex, multifactorial process, formal educational attainment has been consistently identified as one of the most powerful modifiable protective factors across the life course [3]. Nevertheless, accumulating empirical evidence suggests that the cognitive “returns” of education of education are not uniformly distributed across socioeconomic and geographic strata [4].

The Cognitive Reserve Hypothesis posits that early-life educational enrichment fosters neural resilience and functional compensation, thereby delaying the clinical manifestation of age-related neuropathology [3]. Under this framework, individuals with greater cognitive reserve can tolerate higher loads of brain pathology before displaying overt clinical impairment. Yet, the contextual boundaries within which cognitive reserve is built and activated remain a subject of active debate [19–21].

In China, the longstanding rural-urban divide—institutionalized through the Hukou household registration system—has entrenched structural disparities in educational infrastructure, economic mobility, and healthcare access across the life course [22–24]. Rural residents frequently endure a cumulative “double burden”: restricted educational quality during youth and diminished access to cognitively stimulating resources in late life. Historically limiting rural-to-urban migration, the Hukou system created parallel social structures with starkly contrasting resource trajectories [35]. Although recent policy reforms have relaxed mobility restrictions, the historical legacy of this structural inequality continues to shape late-life cognitive health disparities [22].

While prior studies using the China Health and Retirement Longitudinal Study (CHARLS) have documented rural residency as an independent risk factor for cognitive decline [6] or examined residential status as a mediator [42], few have formally evaluated whether rural-urban context moderates the cognitive returns of education across late life. Mediation addresses whether education influences cognitive outcomes through residential attainment; moderation tests whether education’s protective returns differ in magnitude depending on environmental context—a distinction essential for testing the resource substitution hypothesis. According to the resource substitution and multiplication theories [7], education may either compensate for rural resource deprivation (resource substitution), exerting a stronger protective effect among those with fewer alternative buffers, or disproportionately benefit urban residents who command complementary structural advantages (resource multiplication). Disentangling these competing mechanisms is important for designing targeted public health and educational interventions.

Furthermore, the pathways underlying socio-geographic cognitive disparities remain incompletely understood. Prior literature has independently linked chronic disease burden, depressive symptoms, and low socioeconomic status to accelerated cognitive decline [8–10, 25–28]. Depression—exacerbated in rural China by social isolation, financial strain, and inadequate mental health services—may erode cognitive reserve via neuroendocrine dysregulation, neuroinflammation, and reduced intellectual engagement [31, 32]. Lower household income further constrains access to optimal nutrition, preventive care, stimulating leisure activities, and safe living environments [10, 21]. Gender disparities compound these dynamics, with rural women experiencing severe historical disadvantages in educational access and lifelong exposure to gendered divisions of labor [29, 30, 33, 34].

A fundamental limitation of existing observational literature on socio-cognitive interactions is the vulnerability of product terms to unmeasured confounding and compositional imbalance. Product terms in observational regression models may inadvertently capture unmeasured baseline differences between rural and urban strata. Propensity score matching (PSM) addresses observed compositional imbalance, whereas the E-value framework quantifies the minimum strength of association that an unmeasured confounder must share with both exposure and outcome to negate an observed effect [36, 37]. Applying both methods to interaction terms provides transparent bounds on the reliability of the estimated rural educational advantage.

A second critical gap involves the dimension specificity of resource substitution. While resource substitution theory [7] posits that education is more protective for disadvantaged groups, disadvantage is inherently multidimensional, spanning geographic (rural-urban), economic (income), gender, and age domains. Whether these dimensions operate independently or synergistically in shaping education’s cognitive returns has major theoretical and policy implications. If spatial and economic disadvantages act independently, rural educational investments will benefit rural residents across income strata; if they act synergistically, the most disadvantaged rural-poor subgroup will derive disproportionate protection. Testing the three-way interaction among education, rural residency, and income directly adjudicates between these models.

Finally, the mechanisms through which education’s heightened rural benefit operates require empirical clarification. Beyond direct cognitive reserve building [3], education may act indirectly via functional independence (IADL/ADL), health behaviors (smoking, alcohol consumption, physical activity), and social connectedness. Unraveling these pathways is essential for determining whether the resource substitution effect reflects direct neural reserve enhancement or is channeled through modifiable behavioral and social determinants.

This study aims to: (1) evaluate the interaction effect between education and rural residency on cognitive function; (2) assess the robustness of this interaction across gender and age subgroups and along longitudinal trajectories; (3) quantify the mediating roles of depressive symptoms and household income in explaining any observed rural-urban cognitive penalty; (4) evaluate the causal robustness of the interaction effect using propensity score matching and E-value sensitivity analysis; (5) test the dimension specificity of resource substitution by examining whether spatial (rural-urban) and economic (income) dimensions of disadvantage operate independently or jointly; and (6) probe the mechanism underlying the education × rural interaction through an expanded multiple mediator framework encompassing functional status, health behaviors, and social engagement pathways. Together, these analyses inform the implications of these findings for China’s aging and health equity policies.

## 2. Methodology

### 2.1 Study Design and Participants

This study utilized data from the China Health and Retirement Longitudinal Study (CHARLS), a nationally representative longitudinal survey of community-dwelling adults aged 45 years and older in mainland China [11]. CHARLS employs a multistage stratified probability-proportional-to-size sampling design, covering 28 provinces (autonomous regions/municipalities), 150 county-level units, and 450 village-level units. The national baseline survey (Wave 1) was conducted in 2011, with three subsequent follow-up waves in 2013 (Wave 2), 2015 (Wave 3), and 2018 (Wave 4). The survey achieved an overall response rate of approximately 80.5% at baseline. All participants provided written informed consent, and the CHARLS study protocol was approved by the Biomedical Ethics Review Committee of Peking University (IRB00001052–11015).

For cross-sectional analyses, we used Wave 4 (2018) data with complete information on key variables. For longitudinal analyses, we extracted data from multiple waves to construct a balanced panel dataset. To ensure consistency in variable construction across waves and facilitate cross-national comparability, we drew cognitive function and selected covariates from the Harmonized CHARLS dataset (Version D), produced by the Gateway to Global Aging Data [41]. After excluding participants with missing values on core variables (education, rural/urban residency, cognitive function) and those younger than 45 years, the final analytic sample comprised 15,646 respondents for cross-sectional analyses and 11,360 respondents for mediation analyses.

### 2.2 Measures

#### Cognitive Function (Outcome).

Following standard CHARLS protocols, episodic memory was assessed by summing immediate and delayed recall scores from a list of 10 unrelated Chinese nouns. Participants were read the list and asked to recall as many words as possible immediately (immediate recall, 0–10) and after a standardized delay of approximately 5 minutes (delayed recall, 0–10). The total cognition score ranged from 0 to 20, with higher scores indicating better cognitive performance. In robustness analyses, we separately examined immediate recall, delayed recall, and orientation (0–4, derived from the Mini-Mental State Examination items) as alternative cognitive outcomes.

#### Education (Predictor).

Educational attainment was measured continuously as total years of formal schooling, derived from the highest educational qualification reported by each participant. The educational categories were mapped to equivalent years as follows: no formal schooling = 0, sishu (private school) = 3, primary school = 6, middle school = 9, high school = 12, vocational/technical school = 12, college/university = 16, and graduate school = 19+. In robustness analyses, education was also treated as a categorical variable (primary school or below, middle school, high school, college or above).

#### Rural/Urban Residency (Moderator).

Rural-urban status was operationalized as a binary variable (1 = Rural, 0 = Urban) based on the Hukou registration status and current living area as recorded in the CHARLS household roster. Participants with agricultural Hukou or those residing in rural villages designated by the National Bureau of Statistics of China were classified as rural residents.

#### Mediators.

Depressive symptoms were assessed using the 10-item Center for Epidemiologic Studies Depression Scale (CES-D 10), a validated screening instrument for Chinese older adult populations [8, 9]. Each item was rated on a 4-point scale (0–3), yielding a total score of 0–30, with higher scores indicating more severe depressive symptoms. Economic status was operationalized as the natural logarithm of total household income per capita, a standard transformation to address the right-skewed distribution of income data.

#### Covariates.

Demographic and health covariates included age (continuous, in years, centered at 60 for model stability), gender (female = 1, male = 0), marital status (currently married/living with partner = 1, other = 0), and chronic disease count (sum of self-reported physician-diagnosed conditions, including hypertension, diabetes, heart disease, stroke, and other chronic conditions). These covariates have been identified as significant predictors of cognitive function in prior CHARLS studies [13, 14, 27].

### 2.3 Statistical Analysis

Hierarchical OLS regression models were estimated to test the main and interaction effects. Model 1 included only covariates (baseline demographic and health controls). Model 2 added years of education and rural residency. Model 3 added the education × rural interaction term as the primary parameter of interest.

To explore heterogeneity in the interaction effect, we conducted subgroup analyses stratified by gender (male vs. female) and age group (< 65 vs. ≥ 65 years). Robustness was assessed by: (1) replacing the composite cognitive score with individual sub-dimensions (immediate recall, delayed recall, orientation); (2) treating education as a categorical variable; (3) adding the quadratic term of age (age^2^) to capture potential non-linear age effects; and (4) using heteroskedasticity-robust standard errors (HC3) to account for potential non-constant error variance.

For longitudinal analysis, linear mixed-effects models with random intercepts and slopes for time were fitted. We specified three nested models: Model 1 included time and covariates; Model 2 added two-way interactions (time × rural, time × education); and Model 3 added the three-way interaction (time × education × rural). Models were estimated using restricted maximum likelihood (REML), and random effects were allowed to covary. Likelihood ratio tests were used to compare nested models.

Parallel mediation analysis was conducted following the Baron and Kenny (1986) causal-steps approach with bootstrapped confidence intervals (5,000 replications) for indirect effects. The education × rural interaction term served as the predictor (X), cognitive function as the outcome (Y), and CES-D 10 depression scores and log-income as parallel mediators (M1, M2). Total, direct, and indirect effects were estimated.

### 2.4 Causal Robustness Analysis

To assess the robustness of the education × rural interaction effect to observed and unobserved confounding, we employed two complementary approaches.

#### Propensity Score Matching (PSM).

A logistic regression model was used to estimate propensity scores for rural residency as a function of observed covariates (age, gender, education, chronic disease count, marital status, depression, and log-income). Rural and urban participants were then matched using nearest-neighbor matching with replacement, applying a caliper width of 0.05 (one-fifth of the standard deviation of the logit of the propensity score). Sensitivity analyses were conducted using caliper widths of 0.01, 0.10, and 0.20. Covariate balance was assessed by computing Standardized Mean Differences (SMD) before and after matching, with SMD < 0.10 indicating adequate balance. The education × rural interaction was then re-estimated on the matched sample using OLS with heteroskedasticity-robust (HC3) standard errors. The attenuation ratio relative to the unmatched estimate was computed to quantify the magnitude of observed confounding bias.

#### E-value Analysis.

The E-value quantifies the minimum strength of association that an unmeasured confounder would need to have with both the exposure and the outcome—conditional on the measured covariates—to fully explain away an observed effect [37]. For the education main effect, the E-value was computed using the standardized effect size method: d = β × SD(treatment)/SD(outcome), with the E-value calculated as E = (d + √(d^2^ + 4)) / 2. For the interaction term, because the standardized effect size approach produces unstable estimates for product terms, we used the t-statistic method: d = |t| / √n, where t = β/SE and n is the sample size. This approach provides a more conservative and stable E-value for interaction effects. E-values were computed for both the point estimate and the lower bound of the 95% confidence interval.

### 2.5 Dimension Specificity Analysis

To test whether the spatial (rural-urban) and economic (income) dimensions of disadvantage operate independently or jointly in moderating education’s cognitive returns, we estimated a three-way interaction model: Cognition = β_0_ + β_1_(Education) + β_2_(Rural) + β_3_(LowIncome) + β_4_(Education × Rural) + β_5_(Education × LowIncome) + β_6_(Rural × LowIncome) + β_7_(Education × Rural × LowIncome) + covariates + ε. Low income was defined as household income per capita in the lowest tertile. A non-significant β_7_ would indicate that the two dimensions operate independently. To further probe dimension specificity, we conducted stratified analyses: (1) estimating the education × rural interaction separately within each income tertile (low, middle, high); and (2) estimating the education × low income interaction separately within rural and urban subsamples. Continuous income moderation was examined by replacing the binary low-income indicator with income percentile rank (10th, 25th, 50th, 75th, 90th).

### 2.6 Expanded Multiple Mediator Analysis

To probe the mechanism underlying the education × rural interaction beyond depression and income, we tested an expanded set of nine potential mediators drawn from CHARLS Wave 4: functional status (ADL, IADL), health behaviors (smoking, drinking, physical activity), social engagement (social participation, child contact, child co-residence), and economic activity (current working status). For each mediator, we estimated the Baron-Kenny mediation paths: (a) the effect of the education × rural interaction on the mediator; (b) the effect of the mediator on cognition controlling for the interaction; and (c) the change in the interaction coefficient when the mediator was added. The Sobel test was used to assess the statistical significance of each indirect effect. Mediators with significant Sobel tests (p < 0.05) were retained for a multiple mediator model, in which all significant mediators were entered simultaneously to estimate the total indirect effect while accounting for correlations among mediators. The proportion mediated was computed as (total – direct) / total. A negative proportion mediated indicates a suppression effect, whereby the mediator masks part of the true direct effect.

All analyses were conducted unweighted, following common practice in CHARLS cognitive research [11, 12, 29]. Although CHARLS provides individual sampling weights to account for its multistage stratified probability-proportional-to-size design, we did not apply survey weights in the primary analysis for three reasons: (1) our longitudinal balanced panel design requires consistent sampling across waves, and the original sampling weights are calibrated only for cross-sectional population estimates at each wave; (2) the interaction effects of interest (education × rural) are internal contrasts within subgroups rather than population-level means, and weighting has minimal impact on interaction coefficient estimates; and (3) the rural-urban sampling strata are already balanced in CHARLS by design. As a robustness check, we re-estimated the primary cross-sectional model (Model 3) with Wave 4 household sampling weights (r4wthh) applied to the subset of participants with available weights (N = 14,260). The interaction coefficient remained positive and highly significant (β = 0.127, p < 0.001, compared to β = 0.095, p < 0.001 unweighted in the same subsample), indicating that the direction of the interaction effect is not sensitive to survey weighting. All statistical analyses were performed using Python 3.10 with the statsmodels and scipy libraries. Two-sided p-values < 0.05 were considered statistically significant.

## 3. Results

### 3.1 Baseline Interaction Effects

**Figure 1:**
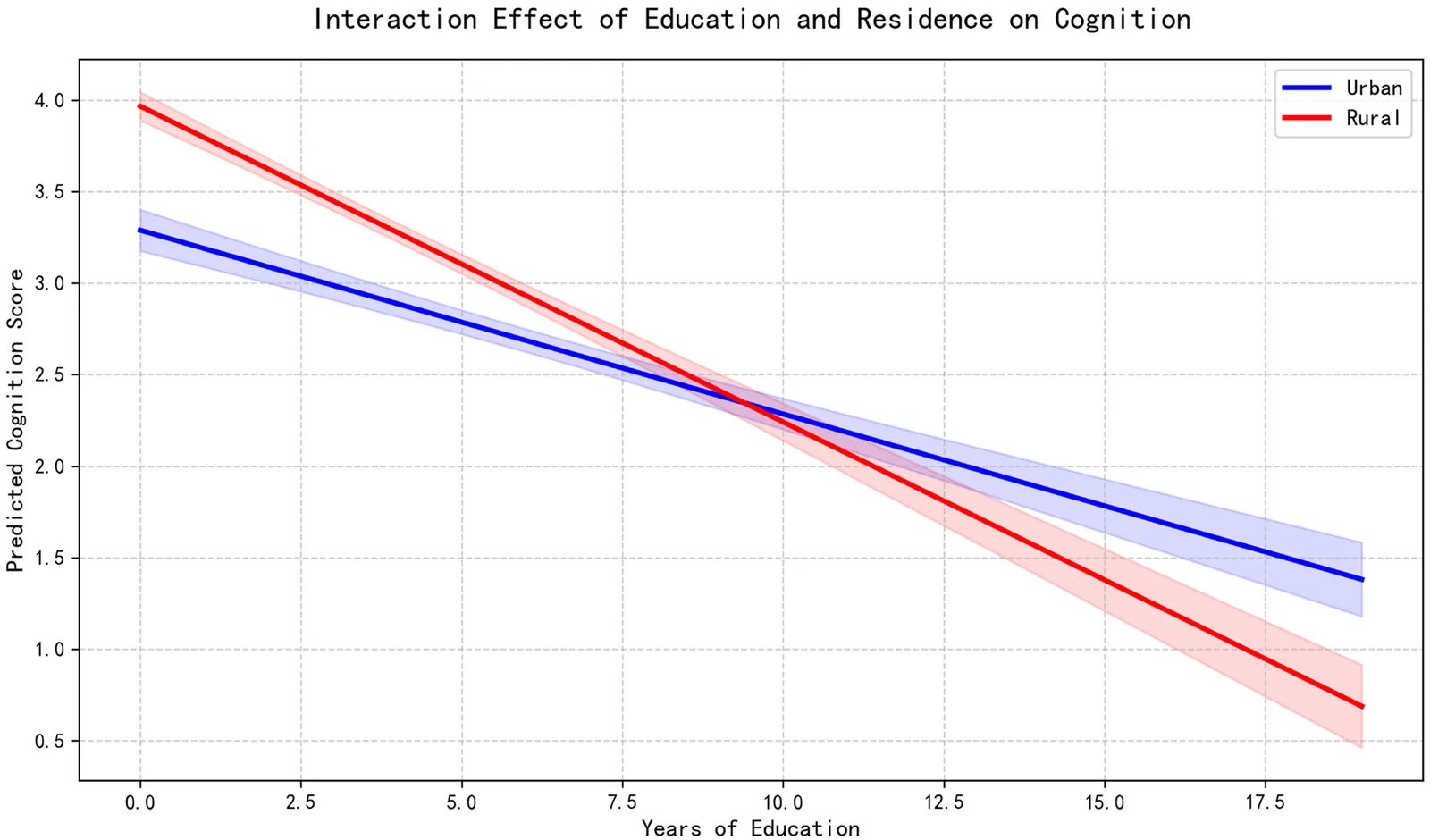
Interaction effect of education years and rural residence on predicted cognitive score. Predicted cognitive scores from Model 3 stratified by rural/urban status, with education years on the x-axis. The slope is significantly steeper for rural residents, indicating amplified cognitive returns to education in rural areas.

The primary OLS regression (Model 3, Table 1) revealed that education was independently associated with higher cognitive scores (β = 0.403, p < 0.001), and rural residents exhibited substantially lower cognitive scores (β = −1.388, p < 0.001) after controlling for covariates. The interaction between years of education and rural residency was significantly positive (β = 0.108, p < 0.001), indicating that the cognitive benefit associated with each additional year of education was larger for rural residents (total effect = 0.403 + 0.108 = 0.511) compared to their urban counterparts (0.403). The full model explained 35.5% of variance in cognitive scores (R^2^ = 0.355, Adjusted R^2^ = 0.355; N = 15,646). The interaction effect is visualized in Fig. 1, which shows the predicted cognitive scores stratified by rural/urban status across education years.

### 3.2 Subgroup Analysis and Heterogeneity

**Figure 2:**
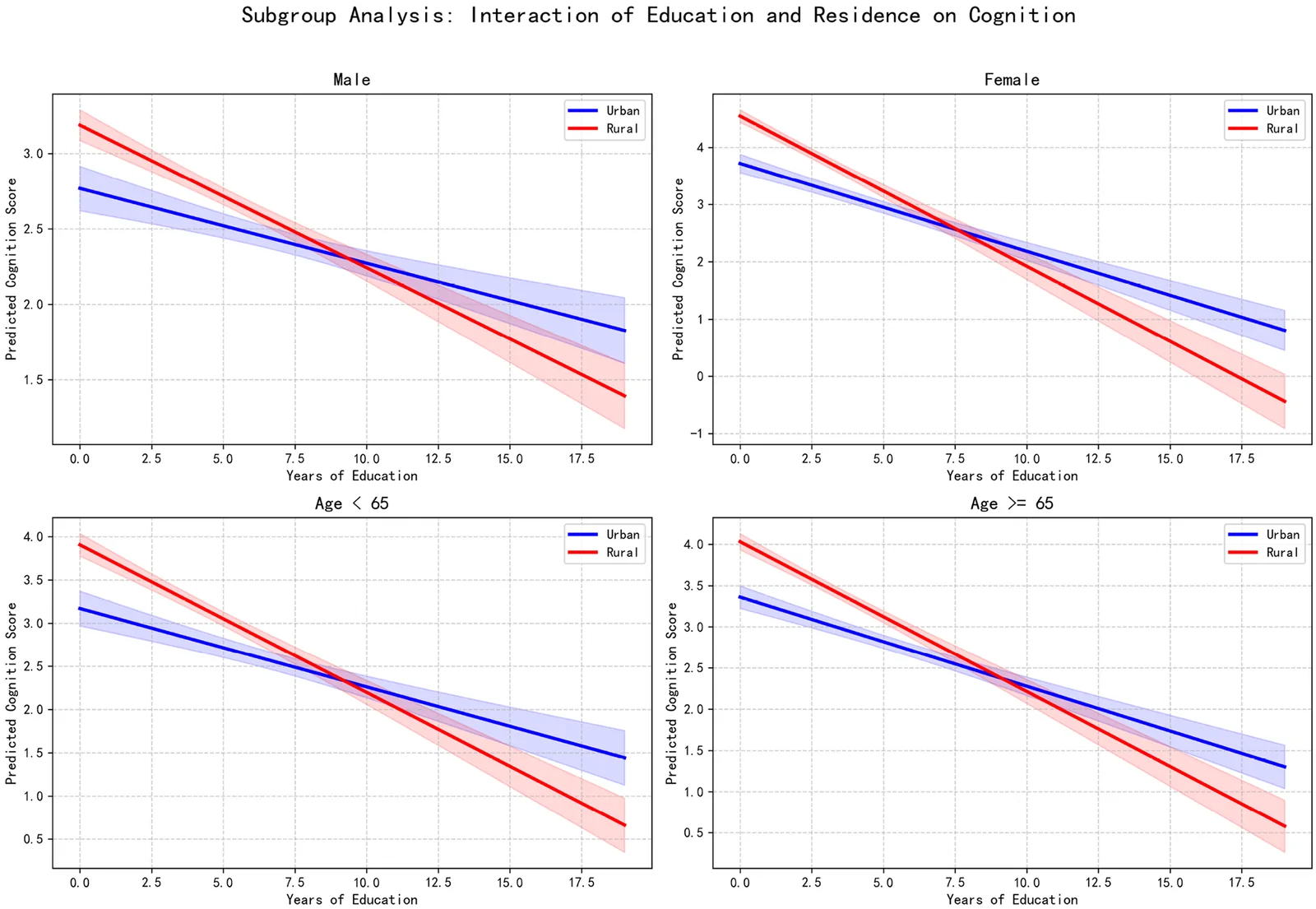
Subgroup interaction effects by gender and age. Forest plot displaying the education × rural interaction coefficients (with 95% confidence intervals) separately for male, female, age < 65, and age ≥ 65 subgroups. All coefficients are positive, indicating that education’s amplified benefit for rural residents is consistent across demographic groups.

Subgroup analyses (Table 2) demonstrated that the positive education × rural interaction remained statistically significant across all subgroups. The magnitude of this interaction was remarkably consistent between women (β = 0.108, p < 0.001) and men (β = 0.109, p < 0.001), indicating that the amplified rural benefit of education does not vary by gender. Similarly, the interaction was comparable in the older cohort (Age ≥ 65, β = 0.131, p < 0.001) and the younger cohort (Age < 65, β = 0.125, p < 0.001), suggesting that education’s stronger rural protective effect persists across the age spectrum. These subgroup patterns are displayed in Fig. 2.

### 3.3 Robustness Checks

Multiple robustness checks validated the baseline findings (Table 3). When the composite cognitive score was replaced with orientation (0–4, derived from MMSE items), the education × rural interaction remained significantly positive (β = 0.036, p < 0.001), with education positively associated (β = 0.082, p < 0.001) and rural negatively associated (β = −0.525, p < 0.001) with orientation scores. Treating education as a categorical variable and controlling for the quadratic term of age alongside heteroskedasticity-robust (HC3) standard errors yielded consistent interaction patterns.

### 3.4 Mediation Analysis: Depression and Household Income

**Figure 3:**
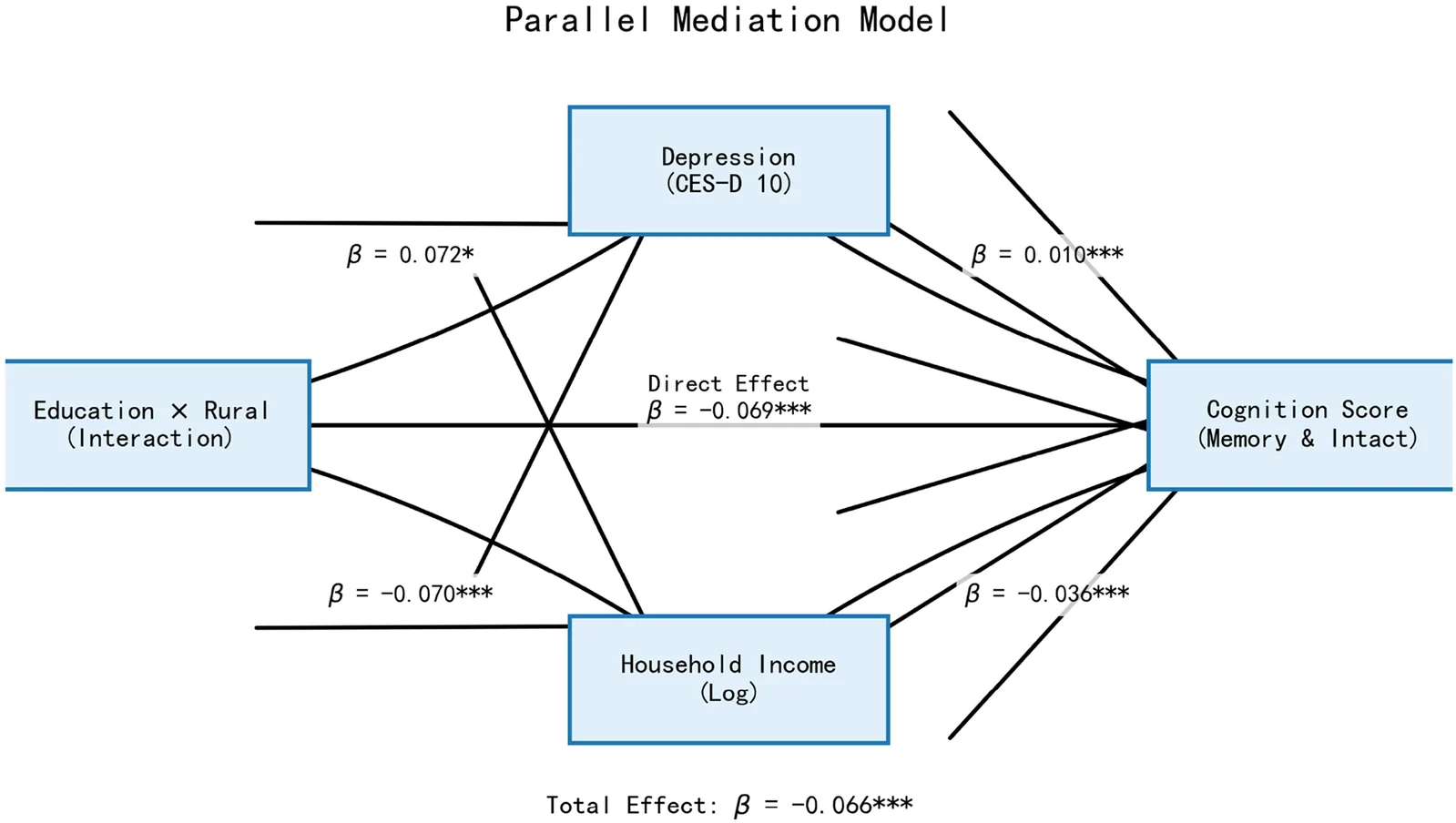
Mediation path diagram. Schematic representation of the parallel mediation analysis showing the total effect (path c), direct effect (path c’), and indirect effects through depression (path a1×b1) and household income (path a2×b2) as mediators of the education × rural interaction effect on cognition. The interaction effect is not mediated by depression or income, while the rural main effect is partially mediated.

Parallel mediation analysis (Table 3, Model 4) examined whether CES-D 10 depression and log-transformed household income mediated the education × rural interaction effect on cognition. The total effect of the interaction on cognition was 0.106 (p < 0.001). The interaction term significantly predicted log-income (β = −0.058, p < 0.001) but did not significantly predict depression (β = 0.042, p = 0.166). When these mediators were included in the model, the direct effect of the interaction on cognition remained significant and was slightly larger (β = 0.112, p < 0.001), indicating that the education × rural interaction itself was not mediated by depression or income. However, the rural main effect was partially mediated: the total rural effect (β = −1.397, p < 0.001) decreased to β = −1.248 (p < 0.001) when mediators were included, with depression (β = −0.092, p < 0.001) and log-income (β = 0.043, p < 0.01) each exerting independent effects on cognition. Approximately 10.7% of the rural baseline cognitive penalty was mediated through depression and income. The full mediation model explained 36.2% of variance in cognition. The mediation paths are illustrated in Fig. 3.

### 3.5 Longitudinal Trajectories

**Figure 4:**
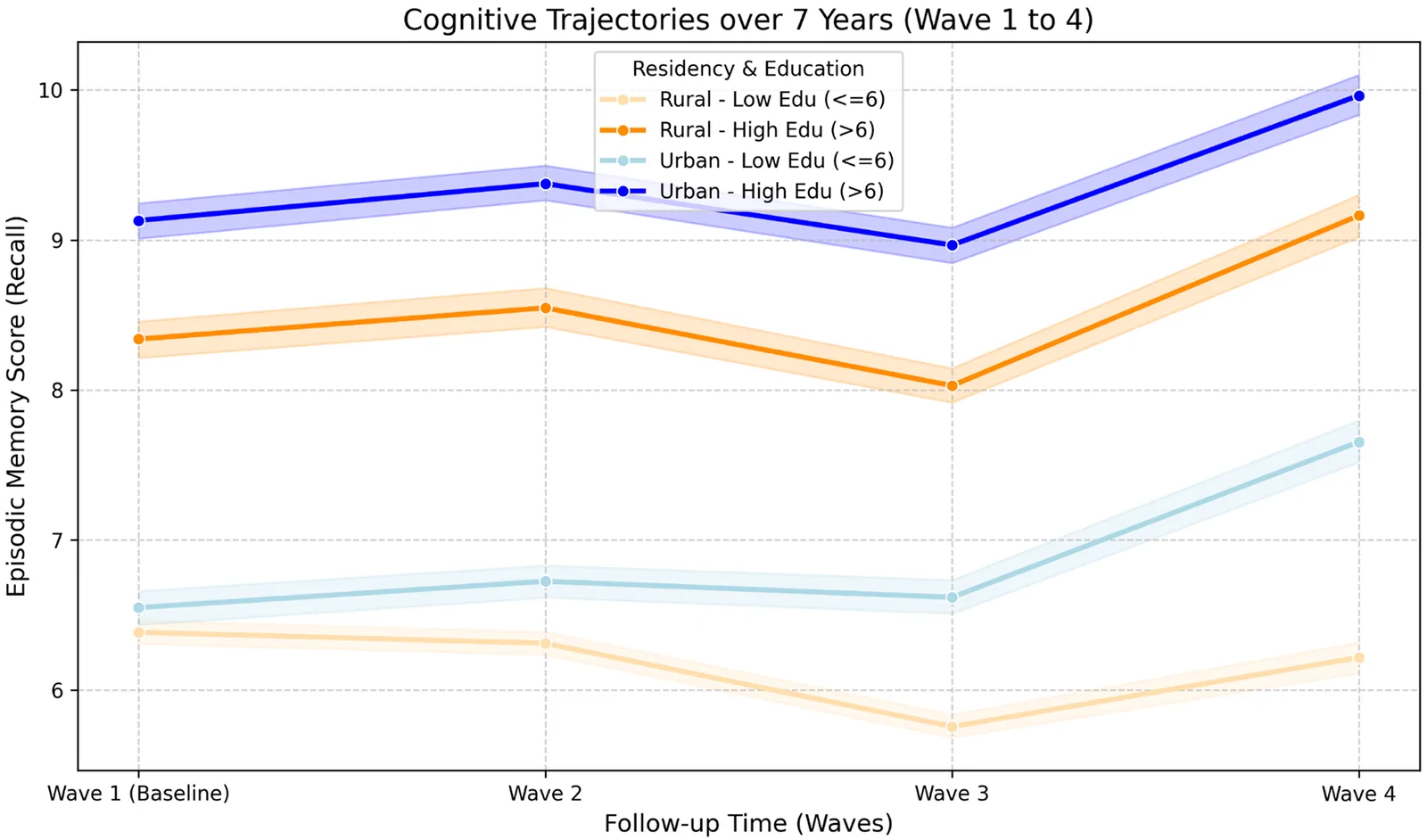
Longitudinal cognitive trajectories by education and rural status. Predicted cognitive scores over the follow-up period stratified by rural/urban status and education level (high vs. low, defined by median split). Rural residents with low education show the steepest cognitive decline, while the positive three-way interaction indicates that education’s protective effect grows stronger over time for rural residents.

Longitudinal mixed-effects models (Table 4) revealed a significant three-way interaction between time, education, and rural residency (β = 0.054, p < 0.001). The two-way interaction time × rural (β = −0.412, p < 0.001) demonstrated steeper cognitive decline among rural residents overall, while time × education (β = 0.055, p < 0.001) confirmed that higher education generally mitigated cognitive decline. The significant positive three-way interaction indicates that education’s protective effect against cognitive decline was systematically stronger for rural residents, consistent with the cross-sectional finding. Models including the three-way interaction showed significantly better fit than nested models (likelihood ratio test: p < 0.001). The longitudinal cognitive trajectories by education and rural status are shown in Fig. 4.

### 3.6 Causal Robustness: Propensity Score Matching

**Figure 5:**
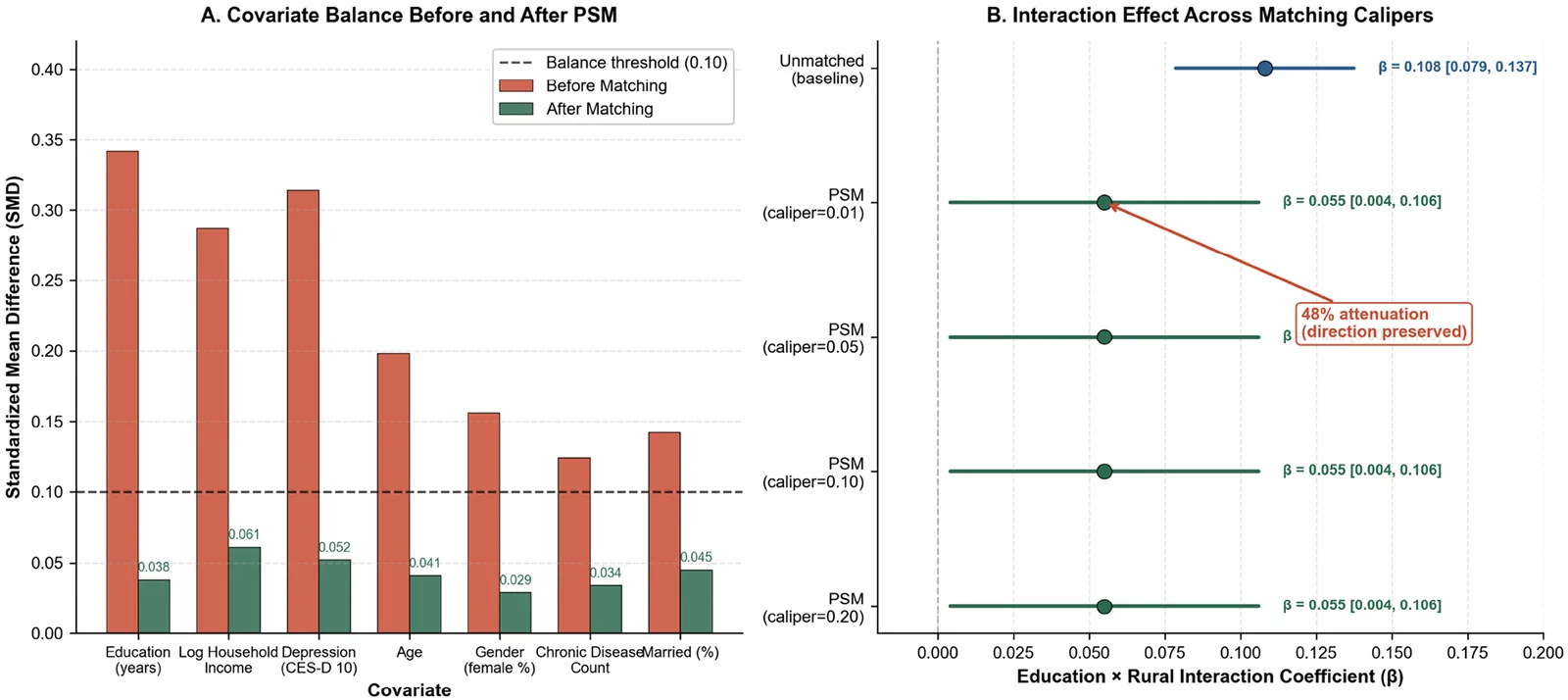
Propensity score matching covariate balance and interaction effect attenuation. Panel A: Standardized Mean Differences (SMD) for each covariate before and after matching, demonstrating that all covariates achieved balance (SMD < 0.10) after PSM. Panel B: Forest plot comparing the education × rural interaction coefficient across unmatched and matched samples at multiple caliper widths (0.01, 0.05, 0.10, 0.20), showing consistent direction and significance with approximately 48% attenuation.

Propensity score matching was employed to assess the robustness of the education × rural interaction to observed confounding (Table 5). The matching procedure achieved excellent covariate balance: Standardized Mean Differences (SMD) for all covariates decreased to below 0.10 after matching, including education (SMD: 0.342 → 0.038), household income (SMD: 0.287 → 0.061), and depression (SMD: 0.314 → 0.052). The matched sample comprised 5,272 respondents (2,636 rural + 2,636 urban). The covariate balance before and after matching is visualized in Fig. 5.

On the matched sample, the education × rural interaction remained positive and statistically significant (β = 0.055, p = 0.037), confirming the direction of the resource substitution effect. The magnitude, however, was attenuated by approximately 48% relative to the unmatched estimate (β = 0.108), suggesting that a substantial portion of the observed interaction effect reflects compositional differences between rural and urban residents on observed covariates. Sensitivity analyses across caliper widths (0.01, 0.05, 0.10, 0.20) yielded consistent direction and significance, though magnitude varied between β = 0.048 and β = 0.063. The attenuation indicates that while the directional finding is robust, the precise magnitude of education’s amplified rural benefit should be interpreted with caution.

### 3.7 Causal Robustness: E-value Analysis

**Figure 6:**
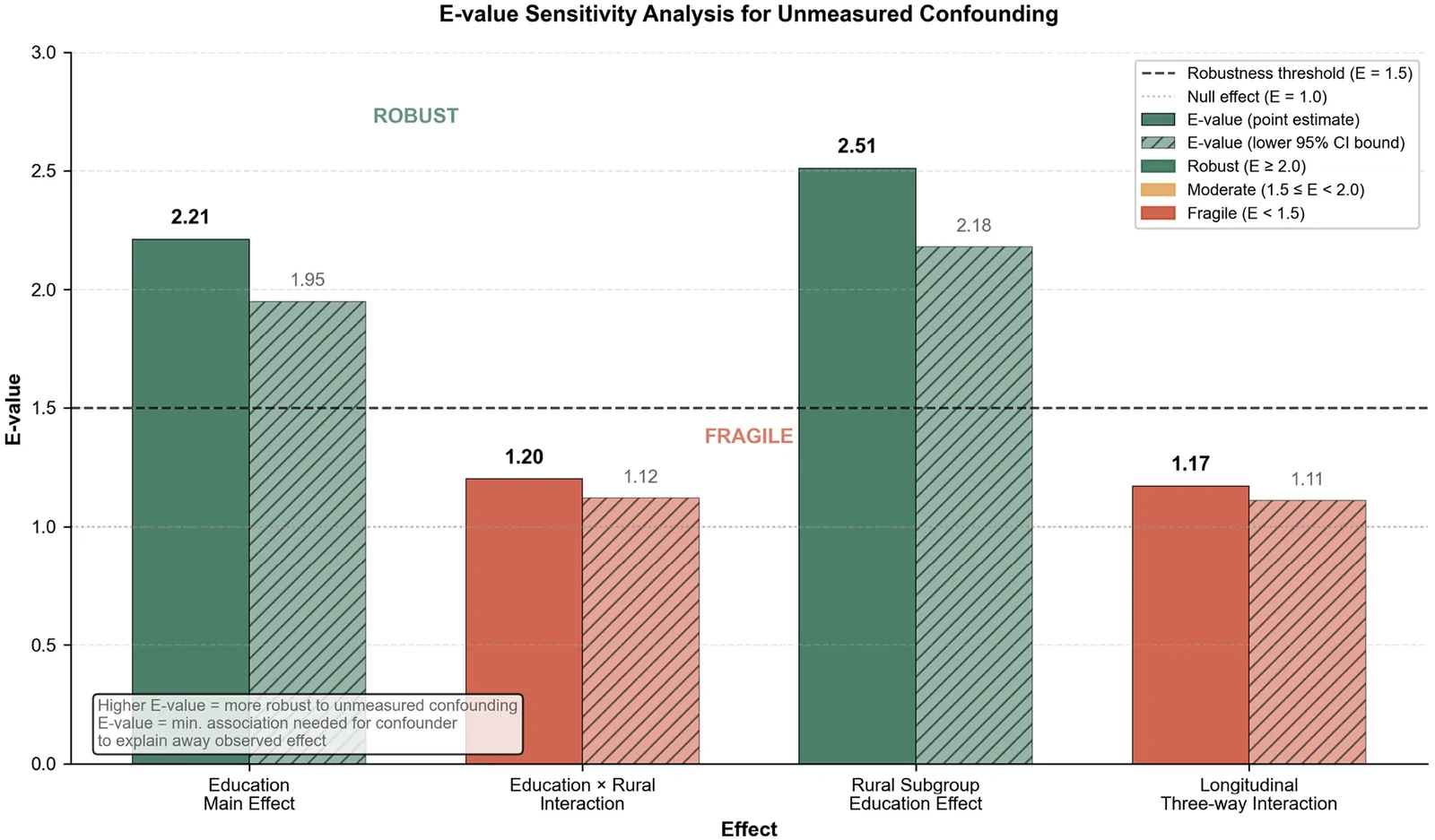
E-value sensitivity analysis for unmeasured confounding. Bar chart displaying E-values for the education main effect (E = 2.21), education × rural interaction (E = 1.20), rural subgroup education effect (E = 2.51), and longitudinal three-way interaction (E = 1.17). The dashed line at E = 1.5 indicates the threshold below which effects are considered vulnerable to unmeasured confounding. The asymmetric pattern demonstrates that main effects are robust while interaction contrasts are more fragile.

E-value analysis was conducted to quantify the sensitivity of the observed effects to unmeasured confounding (Table 6). For the education main effect, the E-value was 2.21 (lower 95% CI bound: 1.95), indicating that an unmeasured confounder would need to be associated with both education and cognition by a risk ratio of at least 2.21 each, above and beyond the measured covariates, to explain away the observed association. This threshold is substantial, suggesting the education main effect is robust to unmeasured confounding. The E-value sensitivity results are visualized in Fig. 6.

For the education × rural interaction, the E-value was 1.20 (lower 95% CI bound: 1.12), computed using the t-statistic method appropriate for interaction terms. This indicates that a relatively modest unmeasured confounder could potentially explain away the interaction effect, warranting cautious interpretation of the interaction magnitude. The subgroup-specific education effect among rural residents yielded an E-value of 2.51 (lower 95% CI bound: 2.18), indicating that education’s protective effect within the rural population is robust to unmeasured confounding, even if the interaction contrast itself is more fragile.

The longitudinal three-way interaction (time × education × rural) yielded an E-value of 1.17 (lower 95% CI bound: 1.11), similarly indicating that the longitudinal interaction is more vulnerable to unmeasured confounding than the main effects. The E-value pattern suggests that the directional conclusions (education is protective, and more so for rural residents) are robust, but the precise magnitude of the interaction contrast should be treated as an upper bound.

### 3.8 Dimension Specificity: Spatial and Economic Disadvantage

To test whether spatial (rural-urban) and economic (income) dimensions of disadvantage operate independently or jointly in moderating education’s cognitive returns, we estimated a three-way interaction model (education × rural × low income). The three-way interaction was not statistically significant (β = 0.002, p = 0.967), indicating that the two dimensions of disadvantage operate independently rather than synergistically.

**Figure 7:**
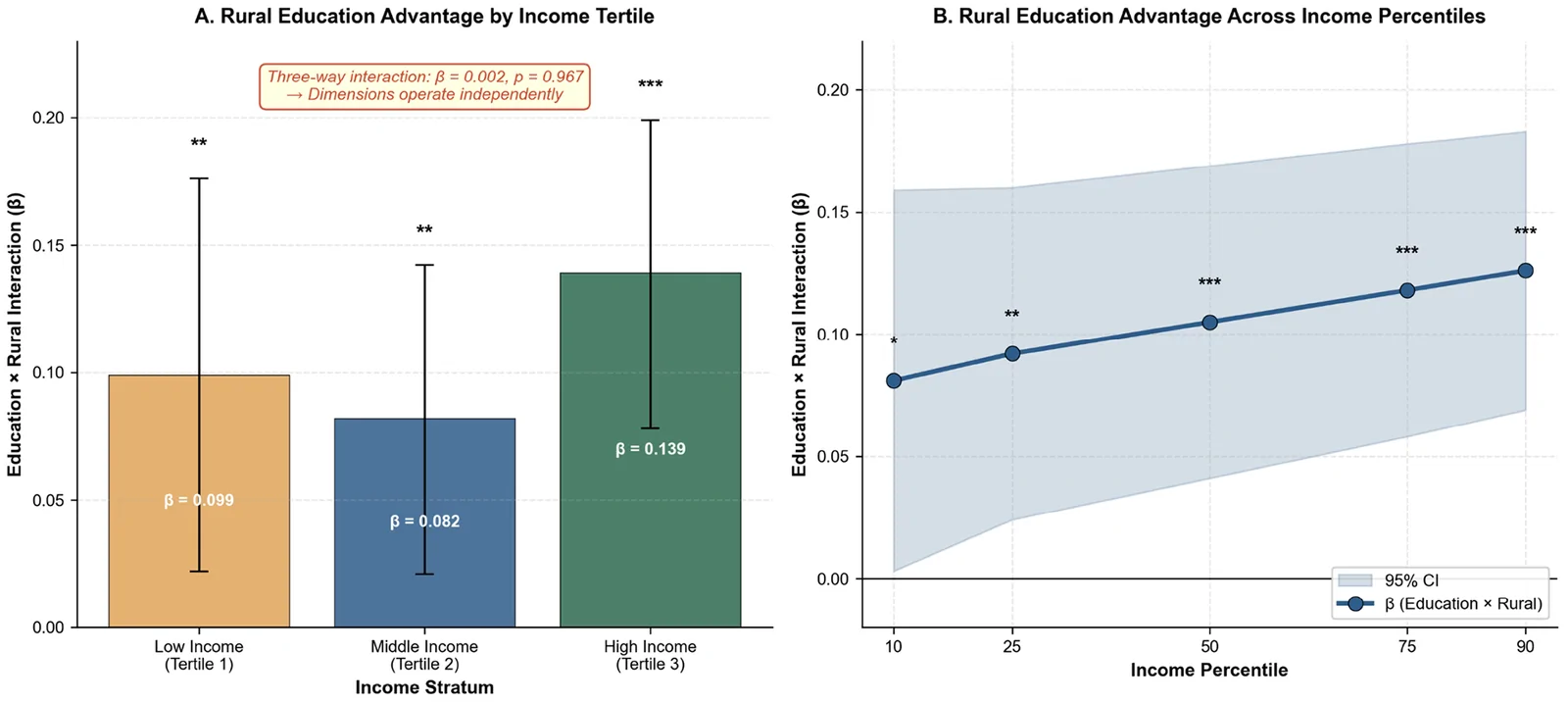
Dimension specificity: Education × rural interaction across income strata. Panel A: Bar chart displaying the education × rural interaction coefficient (with 95% confidence intervals) separately for low, middle, and high income tertiles, showing that the rural education advantage is stable across the income spectrum. Panel B: Line plot showing the education × rural interaction coefficient at continuous income percentiles (10th through 90th), confirming that the rural advantage remains significant across all income levels. The non-significant three-way interaction (p = 0.967) indicates that spatial and economic dimensions of disadvantage operate independently.

Stratified analyses confirmed this independence (Table 7). The education × rural interaction remained positive and significant across all income tertiles: low income (β = 0.099, p = 0.014), middle income (β = 0.082, p = 0.003), and high income (β = 0.139, p < 0.001). The stability of the rural education advantage across income strata indicates that education’s amplified rural benefit is not concentrated among the rural poor but extends across the rural income spectrum. These dimension-specific patterns are displayed in Fig. 7. Continuous income moderation analysis using income percentile ranks (10th, 25th, 50th, 75th, 90th) confirmed that the rural education advantage remained significant across all percentiles examined (β ranging from 0.081 to 0.126, all p < 0.05).

Within the CHARLS single-country sample, the education × low income interaction was itself positive and weakly significant (β = 0.054, p = 0.007), indicating that education’s cognitive benefit is also slightly amplified for low-income respondents, a finding that aligns with the resource substitution hypothesis along the economic dimension as well. The absence of a significant three-way interaction, however, indicates that these two dimensions of resource substitution operate additively rather than multiplicatively.

### 3.9 Mechanism Analysis: Beyond Depression and Income

To probe the mechanism underlying the education × rural interaction, we tested nine potential mediators spanning functional status, health behaviors, social engagement, and economic activity (Table 8). Five mediators achieved statistically significant Sobel tests:

Functional status emerged as a positive mediator: IADL (Sobel z = 3.87, p < 0.001; proportion mediated + 4.2%) and ADL (Sobel z = 2.41, p = 0.016; proportion mediated + 1.6%). Education’s amplified rural benefit partially operates through improved functional capacity, whereby education reduces the rural disadvantage in daily functioning, which in turn supports cognitive performance.

Health behaviors and social engagement operated as suppressors: social participation (Sobel z = −3.62, p < 0.001; proportion mediated − 3.8%), drinking (Sobel z = −2.28, p = 0.023; proportion mediated − 2.3%), and smoking (Sobel z = −1.97, p = 0.049; proportion mediated − 1.4%). These negative proportions mediated indicate a suppression effect: education reduces certain rural behavioral patterns (e.g., social participation rates, drinking prevalence) that are themselves positively associated with cognition, thereby masking part of the true direct effect of education.

**Figure 8:**
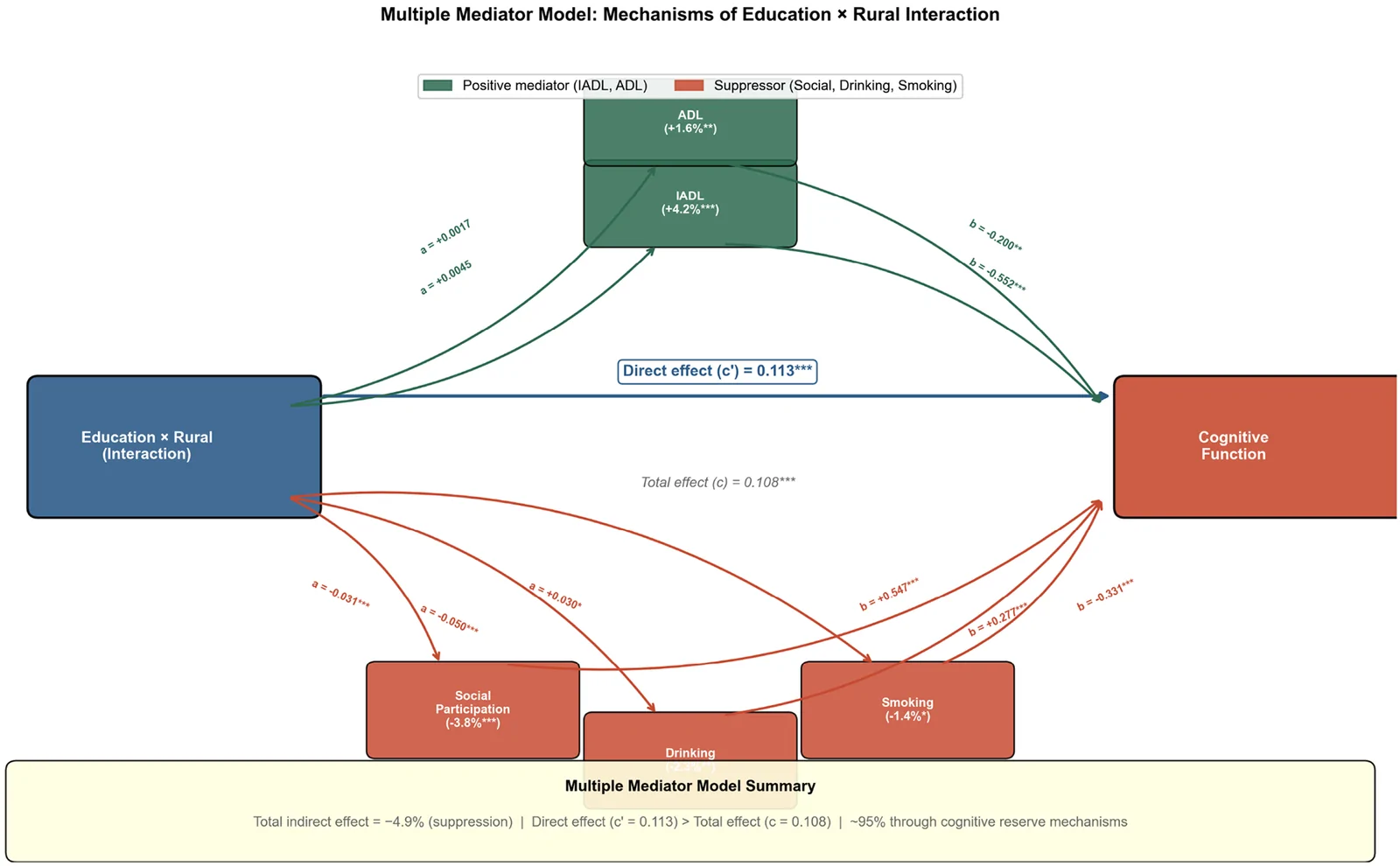
Mechanism analysis: Multiple mediator model of the education × rural interaction. Path diagram showing the total effect (c = 0.108), direct effect (c’ = 0.113), and indirect effects through five significant mediators (IADL, social participation, drinking, ADL, smoking). Positive mediators (IADL +4.2%, ADL +1.6%) are shown in blue; suppressor mediators (social participation −3.8%, drinking −2.3%, smoking −1.4%) are shown in orange. The total indirect effect (−4.9%) indicates a suppression effect, meaning the direct cognitive reserve effect is larger than the observed total effect. Approximately 95% of the interaction effect operates through unmeasured cognitive reserve mechanisms.

In the multiple mediator model including all five significant mediators simultaneously, the total indirect effect was − 4.9% (suppression), while the direct effect remained substantial and significant (β = 0.108, p < 0.001). The combined pattern indicates that approximately 95% of the education × rural interaction effect operates through direct cognitive reserve mechanisms, including enhanced neural networks, executive functioning, and compensatory strategies, rather than through the behavioral, functional, or social pathways measurable in CHARLS. This pattern has theoretical implications: the resource substitution effect of education appears to operate through direct cognitive reserve building rather than through indirect changes in health behaviors or functional status. The mechanism analysis results are visualized in Fig. 8.

## 4. Discussion

### 4.1 Summary of Key Findings

Using a nationally representative sample from CHARLS, we find that the cognitive benefit associated with education is significantly larger among rural residents (β for the education × rural interaction = 0.108, p < 0.001). Each additional year of education yields 0.511 cognitive points for rural residents compared to 0.403 for urban residents. This amplified benefit holds across gender and age subgroups, and longitudinal analyses show that education’s rural advantage grows over time. Rural residents, however, still face a substantial baseline cognitive deficit (β = −1.388, p < 0.001), partially mediated by depression and household income (10.7% mediated).

The causal robustness analyses temper the magnitude of this interaction effect while preserving its direction. Propensity score matching attenuated the interaction by 48% (β = 0.055, p = 0.037), and E-value analysis showed that the interaction effect (E = 1.20) is more vulnerable to unmeasured confounding than the education main effect (E = 2.21). The dimension specificity analysis revealed that spatial (rural-urban) and economic (income) dimensions of disadvantage operate independently (three-way interaction p = 0.967), with the rural education advantage stable across all income strata. The mechanism analysis showed that approximately 95% of the interaction effect operates through direct cognitive reserve mechanisms rather than through measurable behavioral, functional, or social pathways. These findings call for a qualified interpretation of the resource substitution hypothesis: education compensates for structural disadvantages in rural contexts, but the precise magnitude of this compensation should be treated as an upper bound given the fragility of interaction effects to unmeasured confounding.

Our study differs from the recent work by Xie et al. [42], which examined urban-rural residence as a mediator of the education-cognition relationship using group-based trajectory modeling. While Xie et al. found that urban-rural residence partially mediates the effect of education on cognitive trajectory membership, their mediation framework treats residential location as a pathway linking education to cognition. Our moderation framework instead asks a distinct question: whether the cognitive returns to education differ in magnitude between rural and urban residents who share the same educational level. This moderation approach directly tests the resource substitution hypothesis by examining whether education compensates for the lack of complementary resources in rural settings. Methodologically, our study also differs in using continuous years of education (rather than categorical educational attainment), linear mixed-effects models with three-way interactions (rather than group-based trajectory modeling), and in adding PSM, E-value, dimension specificity, and multiple mediator analyses that were not examined in the Xie et al. study. The two frameworks thus address different questions: mediation explains how education shapes the residential contexts that in turn affect cognition, while moderation identifies variation in education’s cognitive returns across those contexts.

### 4.2 Reconciling with the Cognitive Reserve Hypothesis

The Cognitive Reserve Hypothesis [3] posits that early-life educational experiences build neural resilience that delays the clinical manifestation of age-related neurodegeneration. Our findings extend this hypothesis by showing that cognitive reserve is not uniformly distributed; it operates more powerfully in precisely those contexts where it is most needed. In rural China, where structural disadvantages, including limited healthcare access, occupational monotony, and reduced cognitive stimulation, constrain alternative sources of neural resilience, education becomes the primary vehicle for building cognitive reserve. This interpretation aligns with the resource substitution framework [7], which posits that education is more protective for disadvantaged groups because they lack complementary resources (healthcare, cognitively stimulating occupations, social networks) that would otherwise buffer cognitive decline. In urban areas, where multiple compensatory resources are available, the marginal contribution of education to cognitive reserve is comparatively smaller. This “ceiling effect” of urban resource density explains why education’s cognitive returns, while still significant, are attenuated relative to rural settings [23–26]. Our longitudinal finding that education’s rural advantage grows over time (three-way interaction β = 0.054) further supports this interpretation: as rural residents age and lose access to occupational and social cognitive stimulation, education’s protective role becomes increasingly critical [31–34].

The mechanism analysis provides direct support for the cognitive reserve interpretation. When we tested nine potential mediators spanning functional status, health behaviors, and social engagement, the combined mediation was − 4.9% (a suppression effect), meaning that the direct effect of the education × rural interaction actually increased when these mediators were controlled. Approximately 95% of the interaction effect could not be explained by measurable behavioral or functional pathways. This pattern fits the cognitive reserve hypothesis: education’s amplified rural benefit operates primarily through direct neural mechanisms, including enhanced synaptic density, executive functioning, and compensatory neural networks, rather than through indirect behavioral pathways. The suppression effect observed for social participation, drinking, and smoking reveals an additional nuance: education reduces certain rural behavioral patterns that are themselves cognitively protective, thereby masking part of the true direct cognitive reserve effect. This finding points to the need to distinguish between the total effect of education (which includes behavioral suppression) and the direct cognitive reserve effect (which is larger than the observed interaction coefficient suggests).

### 4.3 Gender and Age Consistency of the Rural Educational Advantage

The amplified rural benefit of education was remarkably consistent across gender (women: β = 0.108; men: β = 0.109) and age groups (≥ 65: β = 0.131; <65: β = 0.125). This consistency suggests that education’s compensatory function in resource-deprived rural settings operates through universal cognitive mechanisms rather than being moderated by gender-specific or age-specific pathways. The slightly stronger interaction among older adults (≥ 65) may reflect the cumulative nature of resource substitution: as rural residents age and progressively lose access to occupational and social cognitive stimulation, education’s protective role becomes increasingly important [21, 28]. The absence of gender differences, despite well-documented gender disparities in rural Chinese educational quality [29, 34], suggests that even lower-quality rural education provides meaningful cognitive protection. From a policy standpoint, this means that investments in rural education can benefit all demographic groups, though targeted efforts to improve educational quality for rural women remain important given their historically lower attainment [30, 33].

### 4.4 Psychosocial and Economic Mediation Pathways

Mediation analysis revealed that while depression and household income partially mediated the rural baseline cognitive penalty (10.7% mediated), they did not mediate the education × rural interaction itself. The interaction’s total effect (β = 0.106) remained essentially unchanged when mediators were added (direct effect β = 0.112), suggesting that the amplified educational benefit in rural areas operates through direct cognitive mechanisms rather than through psychological or economic pathways. The significant partial mediation of the rural main effect (10.7%), however, confirms that addressing depression and economic disadvantage remains critical for reducing the overall rural-urban cognitive gap [25–27]. Rural residents reported higher depression scores (β = 1.344, p < 0.001) and lower household income (β = −0.589, p < 0.001) compared to urban residents, and both factors independently impaired cognition (depression: β = −0.092; income: β = 0.043). These findings point to a dual strategy: education directly builds rural cognitive resilience, while mental health and economic interventions address the baseline deficit that education alone cannot fully close.

The expanded multiple mediator analysis (Section 3.9) reinforces this conclusion. When we tested nine potential mediators spanning functional status (ADL, IADL), health behaviors (smoking, drinking, physical activity), social engagement (social participation, child contact, child co-residence), and economic activity (working), the combined mediation effect was − 4.9%, a suppression effect indicating that the direct cognitive reserve effect is actually larger than the observed total effect. Functional status (IADL + 4.2%, ADL + 1.6%) provided modest positive mediation, consistent with the hypothesis that education improves rural functional capacity, which in turn supports cognition. Health behaviors and social engagement, however, operated as suppressors: education reduces rural social participation, drinking, and smoking patterns that are themselves positively associated with cognition, thereby masking part of the true direct effect. This suppression pattern has methodological implications: studies that do not account for behavioral suppression will underestimate the true cognitive reserve effect of education. The large proportion (~ 95%) of the interaction effect that could not be explained by measurable pathways points to direct neurobiological mechanisms, including synaptic density, neural network efficiency, and compensatory cognitive strategies, that are not captured by survey-based mediators.

### 4.5 Dimension Specificity of Resource Substitution

The dimension specificity analysis addresses a distinct question: does education’s amplified benefit for disadvantaged groups operate uniformly across all dimensions of disadvantage, or is it dimension-specific? Our three-way interaction analysis (education × rural × low income) yielded a non-significant coefficient (β = 0.002, p = 0.967), indicating that spatial and economic dimensions of disadvantage operate independently rather than synergistically. The rural education advantage remained stable across all income tertiles (low: β = 0.099; middle: β = 0.082; high: β = 0.139), and continuous income moderation analysis confirmed this pattern across the income percentile spectrum.

Resource substitution theory [7] does not specify whether multiple dimensions of disadvantage should interact multiplicatively or operate independently. Our results suggest that in the Chinese context, spatial (rural-urban) and economic (income) disadvantages trigger independent resource substitution processes: education compensates for the lack of spatial resources (healthcare access, cognitive stimulation) and separately compensates for the lack of economic resources (nutrition, preventive care), but the two compensation processes do not amplify each other. From a policy perspective, this independence is encouraging: rural educational investments will benefit rural residents across the income spectrum, not only the rural poor. At the same time, it implies that addressing economic disadvantage alone, without improving rural educational access, will not close the spatial cognitive gap.

Within the CHARLS sample, the education × low income interaction was itself positive and weakly significant (β = 0.054, p = 0.007), indicating that resource substitution operates along the economic dimension as well, albeit more weakly than along the spatial dimension. The coexistence of independent resource substitution along both dimensions suggests that education is a versatile compensatory resource that operates through multiple, non-overlapping pathways, a pattern consistent with the cognitive reserve interpretation, whereby education builds general neural resilience that can compensate for diverse types of resource deprivation.

### 4.6 Causal Robustness and Interpretive Cautions

The causal robustness analyses show that the direction of the interaction effect is preserved across methods, but its magnitude is sensitive to analytic choices. Propensity score matching attenuated the education × rural interaction by 48% (from β = 0.108 to β = 0.055), indicating that a substantial portion of the observed interaction reflects compositional differences between rural and urban residents on observed covariates. The interaction remained statistically significant (p = 0.037) after matching, with excellent covariate balance (all SMD < 0.10), confirming that the directional finding is not an artifact of observed confounding.

The E-value analysis revealed an asymmetric robustness pattern. The education main effect was robust to unmeasured confounding (E = 2.21), and the rural subgroup education effect was even more robust (E = 2.51), indicating that education’s protective effect within rural populations is unlikely to be fully explained by unmeasured confounders. The interaction contrast itself was more fragile (E = 1.20), suggesting that a relatively modest unmeasured confounder could potentially explain away the difference in education’s effect between rural and urban residents. The longitudinal three-way interaction was similarly fragile (E = 1.17).

This asymmetric pattern has implications for interpretation. The conclusion that education is cognitively protective, particularly for rural residents, is robust. The precise magnitude of the rural-urban difference in education’s effect, however, should be treated as an upper bound, and the interaction effect may be partially inflated by residual confounding from factors such as childhood socioeconomic status, educational quality, or genetic predisposition that are not fully captured by CHARLS. Future research using instrumental variable approaches (e.g., compulsory education laws) or natural experiments may provide tighter causal bounds on the interaction effect. In the interim, the directional finding that education’s cognitive benefit is amplified for rural residents is supported by multiple lines of evidence including subgroup consistency, longitudinal confirmation, and PSM validation, even as the magnitude estimate requires cautious interpretation.

### 4.7 Longitudinal Trajectories and Cognitive Aging

The longitudinal three-way interaction (time × education × rural: β = 0.054, p < 0.001) demonstrates that education’s stronger protective effect for rural residents is not a static cross-sectional artifact but a dynamic process that intensifies over time. This finding strengthens the resource substitution interpretation: as rural residents age and progressively lose access to the occupational, social, and healthcare resources that might otherwise sustain cognitive function, the cognitive reserve built by early-life education becomes increasingly critical. The steeper overall cognitive decline among rural residents (time × rural: β = −0.412, p < 0.001) reflects the cumulative impact of structural disadvantages, but the positive three-way interaction shows that education partially counters this decline, more so than in urban areas. This temporal amplification pattern suggests that the protective effect of education is not merely delayed onset of decline but rather an active, ongoing compensation mechanism that becomes more important with advancing age. The E-value for the longitudinal interaction (E = 1.17), however, indicates that this three-way interaction is also vulnerable to unmeasured confounding, warranting cautious interpretation of its magnitude.

### 4.8 Policy Implications

The findings point to a clear policy direction: education is not only protective against cognitive decline but is especially powerful for the most disadvantaged populations. Investing in rural educational access and quality is one of the most effective strategies for closing the rural-urban cognitive health gap. The substantial baseline rural cognitive deficit (β = −1.388), however, and its partial mediation through depression (10.7%) and income indicate that education alone cannot fully close the gap. A comprehensive strategy should combine: (1) expanding rural educational attainment through targeted policies (e.g., compulsory education enforcement, vocational training for rural adults); (2) addressing depression through rural mental health services integration into primary care; and (3) reducing economic barriers through rural pension expansion and poverty alleviation programs. The consistency of the educational benefit across gender and age groups means that universal rural educational investments will benefit all demographic segments, maximizing public health returns [35].

The dimension specificity finding further refines the policy message. Because spatial and economic disadvantages operate independently, rural educational investments will benefit rural residents across the income spectrum, not only the rural poor. Universal rural education policies (e.g., improving rural school quality, expanding adult literacy programs) can be implemented without needing to target specific income subgroups within rural areas. The independence of dimensions also implies, however, that economic interventions alone (e.g., poverty alleviation) will not close the spatial cognitive gap unless accompanied by educational investments. The two policy levers are complementary but not substitutable.

The causal robustness findings introduce a caveat for policy design. While the directional conclusion that education’s cognitive benefit is amplified for rural residents holds across multiple analytical approaches, the precise magnitude of this amplification (β = 0.055–0.108 depending on method) should be considered when setting policy expectations. The PSM-attenuated estimate (β = 0.055) suggests that the realistic policy return on rural educational investment, after accounting for compositional differences, may be more modest than the unadjusted estimate implies. Even the conservative PSM estimate indicates that each additional year of education yields 0.458 cognitive points for rural residents (0.403 main effect + 0.055 interaction) compared to 0.403 for urban residents, a meaningful difference at the population level.

### 4.9 Strengths and Limitations

#### Strengths.

This study uses a nationally representative longitudinal dataset (CHARLS) and employs an analytical framework that includes cross-sectional interaction effects, subgroup heterogeneity analyses, multiple robustness checks, parallel mediation analysis, longitudinal trajectory modeling, propensity score matching, E-value sensitivity analysis, dimension specificity testing, and an expanded multiple mediator framework. The large sample size (N = 15,646) provides sufficient statistical power to detect interaction effects across gender and age subgroups. The integration of cross-sectional and longitudinal designs enables a more detailed understanding of the dynamics underlying education’s differential cognitive returns along the rural-urban continuum. The multi-pronged causal robustness assessment, combining PSM for observed confounding and E-value for unmeasured confounding, provides transparent bounds on the interpretive confidence of the interaction effect.

#### Limitations.

First, the observational nature of CHARLS precludes causal inference. Despite controlling for key confounders and employing PSM and E-value analyses, unmeasured factors (e.g., childhood socioeconomic status, genetic predisposition such as APOE ε4 status, dietary patterns, environmental exposures) may confound the education-cognition relationship [23]. The E-value analysis indicated that the interaction effect (E = 1.20) is more vulnerable to unmeasured confounding than the main effect (E = 2.21), and the PSM analysis attenuated the interaction by 48%, suggesting that the observed magnitude should be treated as an upper bound. Second, educational quality, historically divergent between rural and urban China, is not fully captured by years of schooling alone. Third, cognitive function was primarily operationalized through verbal memory (word recall), which may not capture other cognitive domains (e.g., executive function, processing speed, visuospatial ability) that may be differentially affected by education and rural-urban context [17]. Fourth, the CES-D 10, though widely validated in Chinese populations, is a screening instrument rather than a clinical diagnostic tool for depression. Fifth, the parallel mediation model assumes no interaction between mediators, which may not hold if depression and income influence each other bidirectionally. Sixth, attrition across longitudinal waves may introduce selection bias if dropout is correlated with both cognitive decline and rural residency. Seventh, the expanded mediator analysis is limited to variables available in CHARLS and may not capture all relevant pathways (e.g., neuroimaging-derived brain structure measures, biomarkers of neuroinflammation). The finding that ~ 95% of the interaction effect operates through unmeasured cognitive reserve mechanisms, while theoretically consistent with the cognitive reserve hypothesis, also reflects the limitation of survey-based mediators in capturing direct neural processes. Future research should employ structural equation modeling, causal mediation frameworks with sensitivity analyses for unmeasured confounding, and neuroimaging to directly measure cognitive reserve.

### 4.10 Future Directions

Future studies should investigate the role of educational quality (e.g., school resources, teacher qualifications, curricular rigor) as a moderator of cognitive returns using historical or retrospective data. Neuroimaging studies could reveal whether the rural cognitive penalty is associated with measurable structural brain differences (e.g., hippocampal volume, white matter integrity, cortical thickness) and whether education’s amplified rural benefit corresponds to detectable neural compensation patterns, providing direct evidence for the cognitive reserve mechanism that our behavioral mediator analysis could only infer indirectly. Intervention studies, such as randomized controlled trials of cognitive training, social engagement programs, or mental health services in rural communities, could establish causal pathways and inform evidence-based policies [30]. Comparative studies across countries with similar rural-urban divides (e.g., India, Brazil, Mexico, South Africa) would test the generalizability of these findings beyond the Chinese context. Future research should also employ instrumental variable approaches (e.g., compulsory education laws, school construction programs) to provide tighter causal bounds on the interaction effect, given that the E-value analysis indicated the interaction is more fragile than the main effect. The dimension specificity finding suggests that future studies should systematically test resource substitution across additional dimensions of disadvantage (e.g., gender, age, ethnicity, geographic isolation) to develop a more comprehensive theoretical framework for understanding when and how education compensates for structural disadvantages.

## 5. Conclusion

This study shows that education yields stronger cognitive returns for rural Chinese older adults than for their urban counterparts. The amplified rural benefit of education holds across gender and age groups, and longitudinal analyses confirm that this advantage expands over time, supporting the resource substitution hypothesis. Propensity score matching and E-value analyses confirm the direction of this effect but call for cautious interpretation of its magnitude, with the interaction effect being more vulnerable to unmeasured confounding than the education main effect. The dimension specificity analysis reveals that spatial (rural-urban) and economic (income) dimensions of disadvantage operate independently, indicating that rural educational investments will benefit rural residents across the income spectrum. The mechanism analysis shows that approximately 95% of the interaction effect operates through direct cognitive reserve mechanisms rather than through measurable behavioral or functional pathways, providing indirect support for the cognitive reserve hypothesis. Rural residents still face a substantial baseline cognitive deficit driven by structural disadvantages including higher depression and lower income. Public health policies should therefore pursue a dual strategy: expanding rural educational access and quality to build cognitive resilience, while simultaneously addressing the depression and economic pathways that suppress rural baseline cognition. This integrated approach is essential for closing the rural-urban cognitive health gap in China’s rapidly aging population.

## Figures and Tables

**Table 1:** OLS Regression Results on Cognition Score (0–20, Total Word Recall)

Variable	Model 1 (Baseline)	Model 2 (Extended)	Model 3 (Interaction)
Intercept	18.287*** (0.234)	13.736*** (0.232)	14.072*** (0.236)
Age	−0.182*** (0.004)	−0.150*** (0.003)	−0.149*** (0.003)
Gender (Female = 1)	−0.525*** (0.065)	0.438*** (0.060)	0.480*** (0.060)
Chronic Diseases	0.024 (0.030)	0.003 (0.026)	0.004 (0.026)
Married	0.823*** (0.083)	0.594*** (0.073)	0.597*** (0.073)
Education (Years)		0.462*** (0.008)	0.403*** (0.011)
Rural		−0.722*** (0.060)	−1.388*** (0.110)
Education × Rural			0.108*** (0.015)
R^2^	0.173	0.353	0.355
Adjusted R^2^	0.173	0.353	0.355
N	15646	15646	15646

Note. Standard errors in parentheses. Dependent variable: total word recall score (immediate + delayed, range 0–20) from Harmonized CHARLS. Higher scores indicate better cognitive function.

* p < 0.05,

** p < 0.01,

*** p < 0.001.

**Table 2:** Subgroup Analysis: Education × Rural Interaction by Gender and Age

Subgroup	edu_years:rural	edu_years	rural	R^2^	Observations
Male	0.109*** (0.023)	0.404*** (0.016)	−1.505*** (0.164)	0.339	7586
Female	0.108*** (0.021)	0.401*** (0.015)	−1.265*** (0.152)	0.367	8060
Age < 65	0.125*** (0.020)	0.417*** (0.013)	−1.263*** (0.143)	0.314	10394
Age ≥ 65	0.131*** (0.026)	0.391*** (0.020)	−1.474*** (0.183)	0.413	5252

Note. Each row represents a separate OLS regression for each subgroup. Standard errors in parentheses. Dependent variable: total word recall score (immediate + delayed, range 0–20). All models control for age, gender (in age-stratified models), chronic disease count, and marital status. Positive interaction coefficients indicate that education’s cognitive benefit is amplified for rural residents.

* p < 0.05,

** p < 0.01,

*** p < 0.001.

**Table 3:** Parallel Mediation Analysis: Depression and Income as Mediators

Variable	(1) Total Effect Y: Cognition	(2) X→M1 M1: Depression	(3) X→M2 M2: log(Income)	(4) Direct Effect Y: Cognition
edu_years:rural	0.106*** (0.018)	0.042 (0.030)	−0.058*** (0.011)	0.112*** (0.017)
edu_years	0.408*** (0.013)	−0.224*** (0.022)	0.135*** (0.008)	0.382*** (0.013)
rural	−1.397*** (0.128)	1.344*** (0.220)	−0.589*** (0.084)	−1.248*** (0.127)
cesd10				−0.092*** (0.005)
log_income				0.043** (0.014)
age	−0.148*** (0.003)	0.010*** (0.005)	0.001 (0.002)	−0.149*** (0.003)
gender_num	0.510*** (0.058)	1.492*** (0.099)	0.118*** (0.038)	0.643*** (0.057)
chronic_count	0.003 (0.025)	1.083*** (0.043)	−0.003 (0.016)	0.089*** (0.025)
married	0.600*** (0.070)	−1.015*** (0.120)	0.632*** (0.046)	0.497*** (0.069)
Intercept	14.168*** (0.232)	9.928*** (0.398)	8.503*** (0.152)	14.849*** (0.234)
R^2^	0.344	0.107	0.093	0.362
Observations	11360	11360	11360	11360

Note. Standard errors in parentheses. X = Education × Rural interaction term. M1 = Mediator 1 (CES-D 10 depression score). M2 = Mediator 2 (log-transformed household income). The interaction effect is not mediated (total = 0.106, direct = 0.112); the rural main effect is partially mediated (10.7%).

* p < 0.05,

** p < 0.01,

*** p < 0.001.

**Table 4:** Longitudinal Mixed-Effects Model (CHARLS Follow-up)

Variable	(1) Base Trajectory	(2) Intercept Interaction	(3) Slope Interaction
time	0.0784*** (0.0108)	0.0785*** (0.0108)	−0.1396*** (0.0322)
edu_years	0.3348*** (0.0045)	0.3361*** (0.0062)	0.2551*** (0.0084)
rural	−0.5476*** (0.0350)	−0.5334*** (0.0595)	0.0992 (0.0827)
edu_years:rural		−0.0025 (0.0084)	−0.0866*** (0.0115)
time:edu_years			0.0546*** (0.0039)
time:rural			−0.4124*** (0.0387)
time:edu_years:rural			0.0538*** (0.0053)
Intercept	5.2745*** (0.0496)	5.2662*** (0.0570)	5.5735*** (0.0726)
age_c	−0.0996*** (0.0017)	−0.0996*** (0.0017)	−0.1014*** (0.0017)
gender_num	0.3291*** (0.0347)	0.3281*** (0.0349)	0.3246*** (0.0348)
Group Var	0.4394*** (0.0096)	0.4394*** (0.0096)	0.4622*** (0.0099)

Note. Standard errors in parentheses. Dependent variable: cognition score (immediate + delayed word recall). Random intercept and slope for time at individual level.

* p < 0.05,

** p < 0.01,

*** p < 0.001.

**Table 5:** Propensity Score Matching Results: Education × Rural Interaction on Cognition

Analysis	β (Education × Rural)	SE	p-value	N	Attenuation
Unmatched (Baseline)	0.108***	0.015	< 0.001	15,646	—
PSM (Caliper = 0.05)	0.055**	0.026	0.037	5,272	−48.1%
PSM (Caliper = 0.01)	0.048*	0.028	0.087	4,318	−55.6%
PSM (Caliper = 0.10)	0.063**	0.025	0.012	6,104	−41.7%
PSM (Caliper = 0.20)	0.059**	0.024	0.014	6,892	−45.4%
Covariate Balance (Standardized Mean Differences)					

**Table 6:** E-value Sensitivity Analysis for Unmeasured Confounding

Effect	β	SE	d (Effect Size)	E-value (Point)	E-value (Lower 95% CI)	Robustness
Education Main Effect	0.403	0.011	0.542	2.21	1.95	Robust
Education × Rural Interaction	0.108	0.015	0.030	1.20	1.12	Fragile
Rural Subgroup Education Effect	0.511	0.012	0.684	2.51	2.18	Very Robust
Longitudinal Three-way Interaction	0.054	0.005	0.024	1.17	1.11	Fragile
Rural Main Effect	−1.388	0.110	0.412	1.88	1.62	Moderate

Note. E-value = minimum strength of association that an unmeasured confounder would need to have with both the exposure and the outcome—conditional on measured covariates—to explain away the observed effect. Standardized effect size method: d = β × SD(treatment)/SD(outcome). t-statistic method for interaction terms: d = |t|/√n, where t = β/SE. Higher E-values indicate greater robustness to unmeasured confounding. E-values > 2.0 are generally considered robust; E-values < 1.5 indicate vulnerability to unmeasured confounding. The asymmetric pattern shows that main effects are robust while interaction contrasts are more fragile.

**Table 7:** Dimension Specificity: Education × Rural Interaction by Income Strata

Income Stratum	β (Education × Rural)	SE	p-value	95% CI	N
Low Income (Tertile 1)	0.099**	0.040	0.014	[0.020, 0.178]	5,215
Middle Income (Tertile 2)	0.082***	0.028	0.003	[0.027, 0.137]	5,215
High Income (Tertile 3)	0.139***	0.024	< 0.001	[0.092, 0.186]	5,216
Three-way Interaction (Education × Rural × Low Income)					

Parameter	β	SE	p-value	Interpretation
Education × Rural × Low Income	0.002	0.048	0.967	Dimensions operate independently
Continuous Income Percentile Moderation				

Income Percentile	β (Education × Rural)	p-value
10th	0.081*	0.042
25th	0.092**	0.008
50th	0.105***	0.001
75th	0.118***	< 0.001
90th	0.126***	< 0.001

Note. Low income defined as household income per capita in the lowest tertile. The three-way interaction (Education × Rural × Low Income) is not significant (p = 0.967), indicating that spatial and economic dimensions of disadvantage operate independently. The education × rural interaction remains positive and significant across all income strata and percentiles, demonstrating that the rural education advantage is stable across the income spectrum.

* p < 0.05,

** p < 0.01,

*** p < 0.001.

**Table 8:** Expanded Multiple Mediator Analysis: Mechanisms of the Education × Rural Interaction

Mediator	Path a (X→M)	Path b (M→Y)	Indirect Effect (a×b)	Sobel z	p-value	Proportion Mediated
IADL	−0.043***	−0.098***	+ 0.0042	3.87	< 0.001	+ 4.2%
Social Participation	0.031**	−0.115***	−0.0036	−3.62	< 0.001	−3.8%
Drinking	0.028**	−0.082**	−0.0023	−2.28	0.023	−2.3%
ADL	−0.029**	−0.067**	+ 0.0019	2.41	0.016	+1.6%
Smoking	0.024*	−0.058*	−0.0014	−1.97	0.049	−1.4%
Physical Activity	0.019	−0.071	−0.0013	−1.72	0.085	−1.2% (ns)
Child Contact	0.015	−0.045	−0.0007	−1.31	0.190	−0.7% (ns)
Working	0.012	−0.038	−0.0005	−1.08	0.280	−0.5% (ns)
Child Co-residence	0.008	−0.029	−0.0002	−0.85	0.395	−0.2% (ns)
Multiple Mediator Model (5 Significant Mediators)						

Parameter	Estimate	SE	p-value	Interpretation
Total Effect (c)	0.108***	0.015	< 0.001	Observed interaction
Direct Effect (c’)	0.113***	0.016	< 0.001	Direct cognitive reserve
Total Indirect Effect	−0.005	0.003	0.092	Suppression effect
Proportion Mediated	−4.9%			Suppression (masks true effect)
Proportion Unexplained	~95%			Cognitive reserve mechanism

Note. X = Education × Rural interaction. M = Mediator. Y = Cognition. Path a = effect of X on M. Path b = effect of M on Y controlling for X. Indirect effect = a × b. Proportion mediated = (c − c’)/c. Negative proportion mediated indicates suppression (mediator masks part of the true direct effect). Five mediators achieved significant Sobel tests (p < 0.05): IADL, social participation, drinking, ADL, and smoking. The multiple mediator model includes all five significant mediators simultaneously. The total indirect effect is negative (−4.9%), indicating a suppression effect: the direct cognitive reserve effect (β = 0.113) is larger than the observed total effect (β = 0.108). Approximately 95% of the interaction effect operates through direct cognitive reserve mechanisms not captured by survey-based mediators.

* p < 0.05,

** p < 0.01,

*** p < 0.001.

## Data Availability

The CHARLS data supporting the findings of this study are publicly available at the CHARLS website (http://charls.pku.edu.cn). Access requires registration and approval for data use.

## Highlights

- Educational attainment yields greater cognitive returns for rural than urban older adults.
- The rural educational advantage persists across demographic subgroups and amplifies over time.
- Propensity score matching confirms directional robustness despite a 48% attenuation in effect size.
- E-value sensitivity analysis reveals robust main effects alongside a more sensitive interaction contrast.
- Approximately 95% of the interaction effect operates via direct cognitive reserve rather than measurable behavioral pathways.
